# Determinants of drug entry into the developing brain

**DOI:** 10.12688/f1000research.20078.1

**Published:** 2019-08-07

**Authors:** Liam Koehn, Mark Habgood, Yifan Huang, Katarzyna Dziegielewska, Norman Saunders

**Affiliations:** 1 Department of Pharmacology & Therapeutics, University of Melbourne, Parkville, Victoria, 3010, Australia

**Keywords:** ABC transporter, Blood-brain barrier, fetus, neonate, cerebrospinal fluid, placenta, permeability

## Abstract

**Background**: A major concern for clinicians in prescribing medications to pregnant women and neonates is the possibility that drugs might have damaging effects, particularly on long-term brain development. Current understanding of drug permeability at placental and blood-brain barriers during development is poor. In adults, ABC transporters limit many drugs from entering the brain; however, little is known about their function during development.

**Methods**: The transfer of clinically relevant doses of paracetamol (acetaminophen), digoxin and cimetidine into the brain and cerebrospinal fluid (CSF) was estimated using radiolabelled drugs in Sprague Dawley rats at three developmental stages: E19, P4 and adult. Drugs were applied intraperitoneally either acutely or following chronic exposure (for five days). Entry into brain, CSF and transfer across the placenta was measured and compared to three markers (L-glucose, sucrose, glycerol) that cross barriers by “passive diffusion”. The expression of ABC transporters in the brain, choroid plexus and placenta was estimated using RT-qPCR.

**Results**: All three drugs entered the developing brain and CSF in higher amounts than the adult brain and CSF. Comparisons with “passive” permeability markers suggested that this might be due to age-related differences in the functional capacity of ABC-efflux mechanisms. In adult animals, chronic treatment reduced digoxin (12% to 5%, p<0.01) and paracetamol (30% to 21%, p<0.05) entry compared to acute treatment, with the decrease in digoxin entry correlating with up-regulation of efflux transporter *abcb1a* (PGP). In fetal and newborn animals, no gene up-regulation or transfer decreases were observed. Instead, chronic paracetamol treatment resulted in increased transfer into the fetal brain (66% to 104%, p<0.001).

**Conclusions**: These results suggest that the developing brain may be more at risk from acute drug exposure than the adult brain due to reduced efflux capacity and at greater risk from chronic treatment due to a lack of efflux mechanism regulatory capacity.

## Abbreviations

ABC, ATP-binding cassette; BCRP, breast cancer resistance protein; CSF, cerebrospinal fluid; DPM, disintegrations per minute; E, embryonic (note that by longstanding convention all gestational ages in rodents are referred to as embryonic, but in this study E19 is a fetal stage); i.p., intraperitoneal; i.v., intravenous; MRP, Multidrug resistance-associated protein; P, postnatal; PGP, P-glycoprotein; RT-qPCR, Real time quantitative polymerase chain reaction; SD, standard deviation; µCi, micro Curie.

## Introduction

The mechanisms that prevent or limit entry of drugs and toxins into the adult brain are reasonably well known. For water-soluble molecules, intercellular transfer is largely prevented by tight-junctions ( Johansson *et al*., 2008; Saunders *et al*., 2008; Saunders *et al*., 2018). For lipid soluble compounds, transcellular transfer of many compounds is limited by efflux transporter mechanisms ( Mandal *et al*., 2017). Members of the ATP-binding cassette (ABC) transporter family, known to be located in the various brain barrier interfaces (Roberts *et al*., 2008; Saidijam *et al*., 2018; Strazielle & Ghersi-Egea, 2015), are major contributors to this protection. They are the main reason why it has proved so difficult to develop new drugs for neurological and neuropsychiatric conditions. It has been estimated that 98% of drugs developed by pharmaceutical companies for such conditions fail to enter the brain in therapeutically useful amounts (Pardridge, 2002).

In contrast to the knowledge about the adult brain, little is known about the presence and functional activity of ABC transporters in the developing brain ( Strazielle & Ghersi-Egea, 2015; Saunders *et al*., 2019). This information is essential for understanding the likelihood of a drug to enter the brain directly from the fetal circulation (once the substance has crossed the placenta) or from the circulation of a newborn (especially pre-term), who lacks placental protection. In the clinic, drugs are administered to pregnant women and newborns for a range of conditions and over 1200 drugs have been prescribed during pregnancy and lactation ( Briggs *et al*., 2017). Evidence of potential harms, except in a few cases, is unclear. The scale of the problem is illustrated by international surveys showing that in all countries studied the proportion of pregnant women who take medications during pregnancy is high ( Wyszynski & Shields, 2016). While the clinical application of these drugs in adults is supported by evidence from clinical trials, such trials have not been conducted in pregnant women and neonates because of obvious ethical concerns ( Lyerly *et al*., 2008). In the absence of these data, doctors have to rely on their experience of observed side effects following the application of drugs to these patient populations. However, in the case of the central nervous system (CNS) the harmful effects may not manifest themselves until much later in baby’s development, making them difficult to track. While controlled clinical trials in these patient populations may remain difficult, enhancing the understanding of barrier permeability and ABC transporter functionality at different developmental stages would provide additional evidence to aid clinicians.

Animal studies on the entry of drugs into the developing brain following administration to pregnant animals or newborns are also very scarce. The few studies that have been published on pregnant rodents have been reviewed in Saunders *et al*. (2019) and will be further considered in the Discussion. To determine the relative contributions of the placental and brain barriers in drug protection of the developing brain, individual measurements in fetal blood and cerebrospinal fluid (CSF) are required. Access of any molecule into the CNS is determined by: (i) their physicochemical properties, such as molecular size and lipid solubility; (ii) biological properties (facilitated transfer by influx mechanism, e.g. glucose, or restricted transfer by efflux mechanisms such as ABC transporters); and (iii) physiological properties of brain barriers that are developmentally regulated (e.g. CSF secretion). The actual level that a molecule reaches in brain and CSF is also influenced by the turnover of CSF (“sink action”, Davson, 1967), which is much less in the developing brain (Saunders, 1992). Transfer of lipid insoluble (hydrophilic) molecules that are passively transferring (i.e. not transported) from blood into the brain and CSF is determined by their molecular size at any stage of brain development ( Dziegielewska *et al*., 1979; Habgood *et al*., 1993). For lipid soluble (hydrophobic) molecules, their permeability is dependent not only on their degree of lipid solubility (octonol/water coefficient or logD _octonol_, Davson, 1967; Rapoport *et al*., 1979) but also on their specificity, if any, for individual ABC transporters that play an active role in molecular exclusion at the barrier. It is this last function that is little understood during brain development but is critical to understand their possible limitation of drug entry into the brain at different stages of its maturation.

A key biological problem, which has implications for understanding potential deleterious effects of drugs administered to pregnant women or new-born infants, is the stage of brain development when the efflux transporters appear and when they become functionally effective. It is not necessarily the case that once a transporter is present it will show the same level of activity throughout development; this might increase or decrease at different times (see Discussion and Ek *et al*., 2010; Møllgård *et al*., 2017).

The present paper describes experiments using three index drugs that are given to pregnant women and/or neonates (paracetamol, digoxin and cimetidine). Two of the drugs selected (digoxin and cimetidine) are used for their peripheral therapeutic effects (cardiovascular and alimentary systems respectively) but there is some evidence that they do enter the brain to a limited extent in adult animals (digoxin: Liu *et al*., 2014; Mayer *et al*., 1997; Taskar *et al*., 2017 and cimetidine: Kodaira *et al*., 2011). There are a few reports of entry of digoxin and cimetidine into the brains in fetal rodents (see Discussion). The third drug, paracetamol, is the most widely used drug during pregnancy ( Werler *et al*., 2005). It is the only analgesic that is regarded as “safe” in infants ( Australian Medicines Handbook, 2019; World Health Organisation, 2012). These three drugs are thought to be substrates for different ABC transporters. There are studies linking PGP ( *abcb1*) to digoxin transfer ( Mayer *et al*., 1996; Smit *et al*., 1999) and BCRP ( *abcg2)* to cimetidine transfer ( Liu *et al.*, 2007; Staud *et al*., 2006). It is unclear from the literature which ABC transporter(s) paracetamol is a substrate for, although it has been suggested that MRP3 ( *abcc3*) may limit paracetamol entry via its glucuronidated metabolite ( Manautou *et al*., 2005). Paracetamol’s other glutathione and sulphate metabolites may be substrates for BCRP ( *abcg2*) or other MRP ( *abcc*) transporters. All three drugs are available in a radiolabelled form. The use of radiolabelled drugs is essential for detection of a drug in the very small volumes of plasma and CSF available from fetal and neonatal rodents.

The results showed that there are clear age-dependent differences in the entry of the three drugs (paracetamol, digoxin, cimetidine) into brain and CSF. In addition, over the course of chronic exposure, drug entry was reduced only after a certain stage of brain maturation. Some of these differences appear to be accounted for by changes in the expression levels of individual ABC transporters. The study provides a basis for future comprehensive research into the entry of a wide range of drugs into the developing brain.

## Methods

### Ethical statement

All procedures involving animals were approved by the University of Melbourne Animal Ethics Committee (Ethics Approval AEC: 1714344.1) and conducted in compliance with Australian National Health and Medical Research Guidelines. All efforts were made to ameliorate any suffering of animals. They were handled by experienced researchers in such a way as to minimise stress prior to being anaesthetised. All animals were assessed as healthy prior to commencement of experiments. Animals were monitored prior to and following every injection ensuring there was no abnormalities in weight (>15%), appearance (wounds, fur) or behaviour (vocalisation, respiration, movements).

### Drug entry studies

***Animals.*** Sprague Dawley rats were supplied by the University of Melbourne Biological Research Facility and housed in groups of 2–4 (adult) or full litters per cage (25cm x 35cm x 25cm on Breeders Choice paper bedding, made from 99% recycled paper; it is biodegradable with no added chemicals), on a 12 h light/dark cycle with *ad libitum* access to food (dry pellets of a fixed formulation diet for laboratory rats and mice fortified with vitamins and minerals to meet the requirements of breeding animals after the diet is autoclaved or irradiated, supplied by Speciality Feeds, Western Australia) and water. Age groups investigated (at treatment completion) were time mated pregnant females at E19 (350-400g body weight), postnatal pups at P4 and non-pregnant female adults (175-230g body weight). E19 was chosen because this is a stage of development when adequate volumes of blood and CSF can be obtained for analysis from fetal rats without pooling ( Dziegielewska *et al*., 1981) and individual pups can be injected intraperitoneally while still inside the uterine horn and kept viable for periods of time. P4 was chosen because its stage of brain development is similar to that of very prematurely born but viable human infants of 22–24 weeks gestation ( Clancy *et al*., 2001). The numbers of animals (n) used for each experiment are indicated in Table 1 and Table 2. Animal numbers were based on previous experience of such experiments and were the minimum number required to detect a significant difference between groups at P <0.05. Animals were selected for treatment groups to ensure weights were statistically similar between direct comparisons.

**Table 1.** Numbers of animals (n) used in the brain and CSF drug permeability experiments. The n numbers listed in brackets are where the numbers of CSF measurements were different from the number of brain measurements. No animal died before the termination of an experiment. E19 and P4 pups were littermates and included both sexes. The differences in pup numbers between groups is due to natural variation in litter sizes. P4 and adult experiments were terminated 30 minutes after tracer administration, whereas the E19 embryo experiments were terminated between 30 minutes and 2.5 hours post-injection due to the time required to sequentially sample each embryo. Acute: experiments involved a single injection of unlabelled drug mixed with a tracer amount of radiolabelled drug. Chronic: animals received multiple drug injections of unlabelled drug over a period of five days (see Methods) with the final injection including the radiolabelled tracer. Adults were non-pregnant females.

Drugs	Permeability		RT-qPCR	
	Acute	Chronic	Acute	Chronic
**Digoxin**				
E19	11 (6)	10	6	6
P4	4	7	6	5
Adult	4	4	4	4
**Cimetidine**				
E19	10 (9)	12 (11)	6	6
P4	5	7	6	6
Adult	4	4	4	4
**Paracetamol**				
E19	11 (7)	9	6	6
P4	4	7	6	6
Adult	4	4	4	4

**Table 2.** Numbers of animals (n) used in “passive” markers permeability experiments. All experiments were acute (single injection including the tracer) with samples collected at 30 minutes post-injection, except for placental transfer experiments where blood samples were collected between 30 minutes to 105 minutes post injection. No animal died before the termination of an experiment. E19 and P4 pups were littermates and included both sexes. Glycerol E19 experiments were conducted in litters from two separate pregnant females. Adults were non-pregnant females.

	Permeability		
Markers	Brain	CSF	Placenta
**Sucrose**			
E19	5	5	-
P4	3	3	
Adult	4	3	
**L-Glucose**			
E19	5	5	12
P4	3	3	
Adult	2	2	
**Glycerol**			
E19	11	11	10
P4	4	3	
Adult	4	4	

***Drug doses.*** Drug doses were selected based on use in clinical practice ( Australian Medicines Handbook, 2019) and adjusted for body weight. Cimetidine (C4522, Sigma-Aldrich) was applied at 11mg/Kg per dose, digoxin (D6003, Sigma-Aldrich) at 30μg/Kg per dose and paracetamol (acetaminophen ≥99.0%, Sigma-Aldrich) at 15mg/Kg per dose.

Cimetidine and paracetamol were dissolved in sterile 0.9% sodium chloride solution.

Digoxin was dissolved in ethanol before dilution in sterile 0.9% sodium chloride solution for injection, with final injectate ethanol concentration <5%. Radiolabelled drugs and hydrophilic markers used are listed in Table 3.

**Table 3.** List of radio-labelled markers, their suppliers and product codes.

Marker	Radiolabel	Molecular weight	Supplier	Code no.
Paracetamol	[2,6- ^3^H]	151	American Radiolabeled Chemicals, Inc.	ART 0679
Digoxin	[ ^3^H(G)]	781	American Radiolabeled Chemicals, Inc.	ART 1323
Cimetidine	[ ^3^H(G)]	252	American Radiolabeled Chemicals, Inc.	ART 1548A
Glycerol	[2- ^3^H]	92	PerkinElmer	NET022L001MC
Sucrose	[U- ^14^C]	342	Amersham International	CFB146
L-glucose	[1- ^14^C]	180	Amersham International	CFA328

***Experimental procedure.*** In acute drug experiments, a single dose was injected that included traces of [ ^3^H]-labelled drug (20µCi adults, 2µCi newborns). In chronic experiments, doses of unlabelled drug were given twice daily (09.00 and 17.00h) for four days before a final injection on the 5 ^th^ day identical to acute experiments. In passive marker experiments, all dosing was acute and contained only the [ ^3^H]- or [ ^14^C]-labelled compound (20µCi adults, 2µCi newborns).

In all experiments involving postnatal animals, injections were intraperitoneal (i.p.). In pregnant animals for fetal drug entry studies, the final injection of the radiolabelled marker was given intravenously (i.v.), as the peritoneal cavity required opening prior to the sampling period to gain access to the fetuses. For passive markers (L-glucose, sucrose and glycerol), fetal animals were individually injected i.p. while still within the uterine horn. All experiments took place between 09.00 and 15.00h. All pregnancy studies were completed with a single treatment group on one day. Postnatal groups were conducted with a single treatment group on one day or with the chronic group completed prior to the acute group.

***Sample collection.*** Samples were collected 30 minutes after the final injection. This duration was chosen partly to limit any potential metabolism of the drugs and markers used but also to allow enough time for i.p. injected markers to reach the blood stream and, from there, to access the brain parenchyma across brain barriers, as well as to limit the CSF sink effect that could influence drug levels ( Bito *et al*., 1966). Thirty minutes is also within the half-life of these drugs ( Adedoyin *et al*., 1987; Harrison & Gibaldi, 1976; Lin & Levy, 1963). The other reason a 30-minute duration was chosen is that in the case of exposed fetal rat pups, it is difficult to maintain them in a reasonable physiological state for long periods due to deterioration of placental perfusion. Therefore, for comparative purposes, all studies were conducted at the same time.

For adult and newborn (P4) experiments, animals were terminally anaesthetized using inhaled isofluorane (IsoFlo 100% w/w, Abbott Laboratories). Blood samples were collected from the right cardiac ventricle, CSF from the cisterna magna (Habgood *et al*., 1992) and cortical segments of brain tissue dorsal to the ventricle of the frontal/parietal lobes, as previously described ( Koehn *et al*., 2019). Blood and CSF sampling from the P4 animals was terminal and would thus not have affected the physiological state of the animals during the experiment.

In pregnancy experiments, animals were anaesthetised i.p. with 25% w/v urethane, Sigma, 1ml per 100g body weight, dose). Animals were placed on a 33°C heating plate in a supine position and an endotracheal catheter inserted to maintain a clear airway. The femoral artery and vein of the left hindlimb were cannulated to provide access for collecting arterial blood samples and intravenous (i.v.) injection of the radio-labelled drug or marker solution; the cannula was flushed with 1ml of heparinized (Hospira Inc, five units per ml) saline. All injections were made by slow infusion. Starting at 30 minutes after injection blood, CSF and brain samples (following terminal exsanguination) were collected serially from each fetus. At the time of each fetus sampling, the state of the placental circulation to the fetus was assessed from the colour of the umbilical blood vessels (pink veins indicate reasonable oxygenation). Time matched blood samples (200μl) were collected from the maternal arterial cannula to establish levels of radioactivity in the maternal circulation at the time of each fetal sampling. Maternal blood volume was maintained by intraarterial injection of equivalent volumes of heparinized sodium chloride solution, which also served to maintain the patency of the cannula. Fetuses were sampled over a period of 30 minutes - 2.5-hours post-injection, the time during which placental circulation to the fetuses remained adequate.

***Sample preparation.*** Samples were processed immediately after collection. Blood was centrifuged at 7000rpm for five minutes and the plasma separated. CSF samples were examined microscopically for traces of red blood cells and discarded if contaminated (Habgood *et al*., 1992). In all experiments, the radioactivity in the injectate was also measured to confirm the uniformity of the injected material. All samples were weighed and transferred into scintillation vials. Soluene350 (0.5ml, PerkinElmer) was added to the brain samples. After being incubated at 36°C overnight to allow the tissue to solubilize, two drops of glacial acetic acid (Sigma) were added to neutralize the strongly alkaline Soluene350. All samples were then mixed with 5ml of scintillation fluid (Emulsifier-safe, PerkinElmer) before being transferred into the liquid scintillation counter (Tri-Carb 4910 TR, PerkinElmer) to count radioactivity disintegrations per minute (DPM) over five minutes each with luminescence correction on. Blank vials containing the same tissues without radioactivity were also counted alongside the samples to establish the level of background counts, which were always subtracted from the radioactivity counts of the corresponding experimental samples.

***Sample analysis.*** Background-corrected DPM data from the liquid scintillation counter were normalized to the weight of the samples and expressed as DPM per µl or µg of sample. Ratios denoting brain and CSF transfer for all animals ( Equation 1) and additional placental transfer between the fetus and the mother for fetal animals ( Equation 2) were obtained:

Equation 1

$Brain\;or\;CSF\;transfer=\frac{Brain\;or\;CSF\;DPM/\mu l}{plasma\;DPM/\mu l}\times 100\%$

Equation 2

$Placental\;transfer=\frac{Fetal\;plasma\;DPM/\mu l}{Maternal\;plasma\;DPM/\mu l}\times 100\%$

### Expression of ABC efflux transporters: RT-qPCR

***Sample collection.*** For RT-qPCR studies, a new set of animals underwent the same experimental procedures as those for the drug entry experiments (above), except that no radioactive tracers were included. Before tissue collection, all surgical instruments were cleaned with RNaseZAP (Thermo Fisher Scientific) to destroy any RNases. For the E19 group, the tissue collected from each pup was brain (see Drug Entry section) and placenta. The small lateral ventricular choroid plexuses were also collected and pooled from all pups into one sample. Tissue collected from P4 and adults (brain and choroid plexus) were processed individually. The samples were placed into sterile cryogenic vials, snap frozen in liquid nitrogen and then transferred into a -80°C freezer for storage.

***RNA extraction and reverse transcription.*** RNA was extracted using RNeasy Plus Mini Kits (QIAGEN, Cat no 74134) for the brain samples in the postnatal animals and E19 placenta samples according to manufacturer’s specifications. For the fetal brain samples and all choroid plexuses, RNA was extracted using RNeasy Micro Kits (QIAGEN, Cat no 74004) according to manufacturer’s specifications. RNA purity and quantity were assessed using a Nano-drop (ND-1000 UV-VIS spectrophotometer, Thermo Scientific) and the concentration of all samples standardized in nuclease-free water (<300ng/μl). Each RNA sample was then converted to cDNA using the High Capacity RNA-cDNA kits (Applied Biosystems, Cat no 4387406) containing 9µl of RNA in Nuclease-free water, 10µl 2x reverse transcriptase buffer mix (RT buffer) and 1µl 20x RT enzyme mix, making a total volume of 20µL. RNA was converted to cDNA using a thermocycler (Veriti 96 Well, Applied Biosystems) set at 35°C for 60 minutes, followed by 95°C for five minutes and then incubated at 4°C until collection. The more stable cDNA was stored at -80°C until ready to analyse with the RT-qPCR system (QuantStudio 6 Flex, Applied Biosystems).

***RT-qPCR.*** Regulation of gene expression was measured using RT-qPCR and amplification detected with SYBR Green fluorescence. Primers listed in Table 4 were made at 800nM, except for *abcc1*, which was at 400nM to avoid reads in no-template control (NTC) wells. PCR efficiencies were validated. The final 10µl reaction volume contained 2µl 1/10 diluted cDNA and 8µL SYBR Green master mix (5µl Rt ^2 ^SYBR Green ROX qPCR master mix, [QIAGEN, Cat no 330529], 1µl each forward and reverse primer, 1µl RNase free water). No-template controls (NTCs) were prepared and analysed alongside the cDNA triplicates with every run. NTCs produced no signal, or the occasional negligible signal (> 38Ct). Plates were evaluated using the Applied Biosystems Quantstudio6 Flex machine: two minutes at 50°C, 10 minutes at 95°C, 40 amplification cycles for 30 seconds at 95°C and one minute at 60°C. Transcript counts were compared to a housekeeper peptidylprolyl isomerase B ( *ppib*) using the equation 2 ^-ΔCt^.

**Table 4.** List of the RT-qPCR primer sequences and the associated NCBI sequences.

Target	Forward Primer	Reverse Primer	NCBI
*ppib*	AGTGACCTTTGGACTCTTTGG	TCCTTGATGACACGATGGAAC	NM_022536.2
*abcb1a*	CAACCAGCATTCTCCATAATA	CCCAAGGATCAGGAACAATA	NM_133401.1
*abcb1b*	CCATGTGGGCAAAGGTACTGA	CTAAGACTTCTTCGGCAACT	NM_012623.2
*abcg2*	CAGCAGGTTACCACTGTGAG	TTCCCCTCTGTTTAACATTACA	NM_181381.2
*abcc1*	CCTTGGGTCTGGTTTACTT	ACAGGGGAACGACTGACAG	NM_022281.2
*abcc2*	CAGGGCTGTGCTTCGAAAATCCAAAA	GTGTGCAGCCTGTGAGCGATGGTGAT	NM_012833.2
*abcc3*	CTCGCCCATCTTCTCCCACTTCTCGG	CCGGTTGGAGGCGATGTAAGGATAAG	NM_080581.1
*abcc4*	GAACGCTACGAGAAAGTCATC	GCCCGTGCCAAGTTCAC	NM_133411.1
*abcc5*	AACAGGAAGGATTCTCAACAGG	TGAATGCTGGACGTGATATGG	NM_053924.1

### Statistical analysis

Group permeability data (brain/plasma and CSF/plasma concentration ratios) for the acute and chronic conditions at all ages are presented as mean ± standard deviation (SD). Microsoft Excel 2011 was used for statistical analysis. Statistical differences between the acute and chronic groups for each drug were determined by unpaired, two-tailed Student’s t-test with p<0.05 accepted as significant and F-tests to confirm equal variance.

## Results

**Figure 1.**
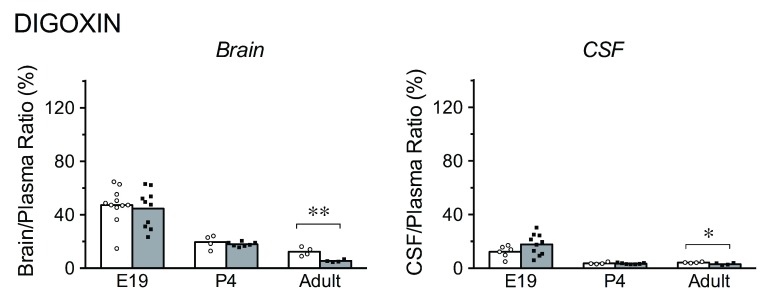
Brain/plasma and cerebrospinal fluid (CSF)/plasma concentration ratios for [ ^3^H]-digoxin in acute (white bars) and chronic (grey bars) experiments. Bars are group means with individual data points shown, * p<0.05, ** p<0.01. Note that for both acute and chronic treatment groups, the ratios are higher in brain and CSF in younger animals. At E19, digoxin was administered by i.p. injection to the mother. Individual fetuses were serially sampled starting at 30 minutes following maternal injection up to approximately 2.5 hours (see Figure 6 for times of sampling and maternal and fetal plasma digoxin levels). Adult and P4 animals were injected i.p. and samples taken at 30 minutes.

The present study investigated the degree of protection that is present at different developmental stages to prevent or limit the transfer of drugs from the circulation into the brain. Rats were injected with either a single dose (acute experiments) or multiple doses (chronic experiments) of one of three drugs (digoxin, cimetidine or paracetamol) and analysed at E19, P4 and adult (see Methods). Results from *in vivo* drug studies were compared with gene expression data to establish which ABC transporters changed their expression following chronic exposure, in order to see if they correlated with observed changes in drug entry results. The transporters studied were: *abcc1-5* (MRP1-5), *abcg2* (BCRP) and *abcb1a/abcb1b* (p-glycoprotein, PGP, which in the rodent has two isoforms). For quality control, the radioactivity counts estimated in different compartments in all experiments are shown in Table 5. The results are then displayed as brain/plasma and CSF/plasma concentration ratios ( Table 6). This representation is a convention used in many blood-brain barrier experiments ( Davson & Segal, 1996); this is because the entry of a marker will depend largely on its amount in circulating blood, which inevitably varies to some extent between experiments (as can be seen from the standard deviations in Table 5). The values in Table 6 and in Figure 1– Figure 3 for each drug represent a measure of “apparent permeability” rather than actual “permeability” because of the influence of the secretion of CSF, which is much lower in the developing brain (Saunders, 1992). Changes in expression levels of eight main ABC transporters in brain (cortex) and lateral choroid plexuses were investigated in chronically treated animals and compared to samples from acute experiments. Throughout this paper, the ABC transporter terminology will reflect the experiment described. For gene-based studies (RT-qPCR), genes will be listed with common protein names in brackets, e.g. *abcc1* (MRP1), whereas for protein-based experimentation, the protein name will be listed with associated gene in brackets, e.g. MRP1 ( *abcc1*). The full raw data are available as *Underlying data* ( Habgood *et al*., 2019).

**Table 5.** Brain (DPM/µg), CSF (DPM/µl) and plasma (DPM/µl) radioactivity levels of individual drugs 30 minutes after administration in adult and P4 animals and 30 minutes - 2.5 hours after administration in E19 embryos. Acute experiments: a single dose of drug mixed with a tracer amount of radiolabelled drug was injected (i.p.). Chronic experiments: twice daily injections (i.p.) over five days of “cold” drug with a tracer amount of radiolabelled drug included in the final dose. Numbers (n) are the same as in Table 1 and indicate samples for brain and plasma, with values in brackets indicating numbers for CSF samples when they differed from those for brain and plasma. Adults were non-pregnant females and littermates of both sexes were included in the E19 and P4 age groups of pups. SD=standard deviation.

		Acute				Chronic			
Digoxin		n	Brain	CSF	Plasma	n	Brain	CSF	Plasma
E19	Mean	11 (6)	8.0	2.3	17.0	10	9.1	3.6	21.0
	SD		2.6	0.9	2.1		2.2	1.6	3.6
P4	Mean	4	23.7	4.4	109.5	7	62.5	12.2	353.2
	SD		6.8	0.5	16.8		16.2	3.6	81.3
Adult	Mean	4	4.5	1.6	36.4	4	2.1	1.3	40.3
	SD		1.2	0.2	5.3		0.8	0.8	19.4
Cimetidine		n	Brain	CSF	Plasma	n	Brain	CSF	Plasma
E19	Mean	10 (9)	7.3	7.0	13.0	12 (11)	8.8	8.4	13.3
	SD		1.7	2.0	2.1		3.0	2.3	2.0
P4	Mean	5	37.2	24.3	310.9	7	45.7	27.8	373.2
	SD		3.8	1.8	11.6		13.0	7.2	73.3
Adult	Mean	4	6.2	6.0	55.9	4	11.6	11.1	90.7
	SD		0.8	1.7	26.1		10.4	10.5	79.7
Paracetamol		n	Brain	CSF	Plasma	n	Brain	CSF	Plasma
E19	Mean	10 (7)	39.6	35.3	57.8	9	146.1	119.0	140.5
	SD		14.2	13.3	12.3		32.5	16.6	15.6
P4	Mean	4	117.5	96.5	193.6	7	84.4	71.0	166.8
	SD		32.4	23.1	27.6		19.2	14.5	39.4
Adult	Mean	4	27.3	25.4	90.1	4	17.6	14.9	83.3
	SD		20.1	15.8	58.7		4.2	4.5	18.5

**Table 6.** Brain/plasma and CSF/plasma concentration ratios (%) for three drugs in acute and chronic experiments at the three developmental ages investigated (mean ± standard deviation). For n numbers see Table 5. Adults were non-pregnant females, E19 and P4 pups of both sexes were littermates respectively.

	Brain/Plasma Ratio		CSF/Plasma Ratio	
Digoxin	Acute	Chronic	Acute	Chronic
E19	47 ± 14	45 ± 14	12 ± 4	18 ± 8
P4	20 ± 5	18 ± 2	4 ± 1	3 ± 1
Adult	12 ± 3	5 ± 1	4 ± 0	3 ± 1
Cimetidine	Acute	Chronic	Acute	Chronic
E19	56 ± 10	65 ± 19	54 ± 11	63 ± 15
P4	12 ± 1	12 ± 2	8 ± 1	7 ± 1
Adult	13 ± 5	13 ± 1	12 ± 3	12 ± 1
Paracetamol	Acute	Chronic	Acute	Chronic
E19	66 ± 18	104 ± 21	60 ± 13	85 ± 10
P4	60 ± 9	51 ± 7	49 ± 6	43 ± 3
Adult	30 ± 4	21 ± 2	29 ± 2	18 ± 2

**Figure 2.**
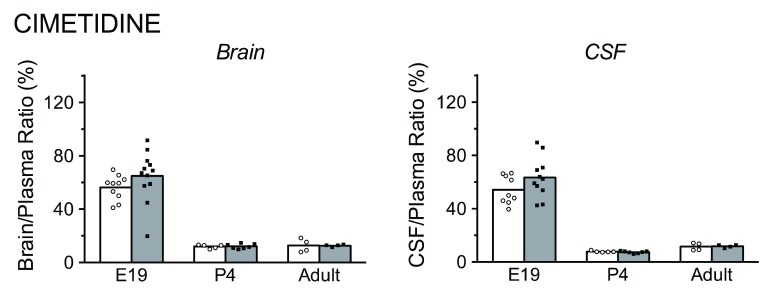
Brain/plasma and cerebrospinal fluid (CSF)/plasma concentration ratios for [ ^3^H]-cimetidine in acute (white bars) and chronic (grey bars) experiments. Bars are group means with individual data points shown. At E19, cimetidine was administered by i.p. injection to the mother. Individual fetuses were serially sampled starting at 30 minutes following maternal injection up to approximately 2.5 hours (see Figure 6 for times of sampling and maternal and fetal plasma cimetidine levels). Adult and P4 animals were injected i.p. and samples taken at 30 minutes. Note that for both acute and chronic treatment groups, the ratios are higher in brain and CSF in younger animals. There were no significant differences between acute and chronic groups at any of the three developmental ages investigated.

**Figure 3.**
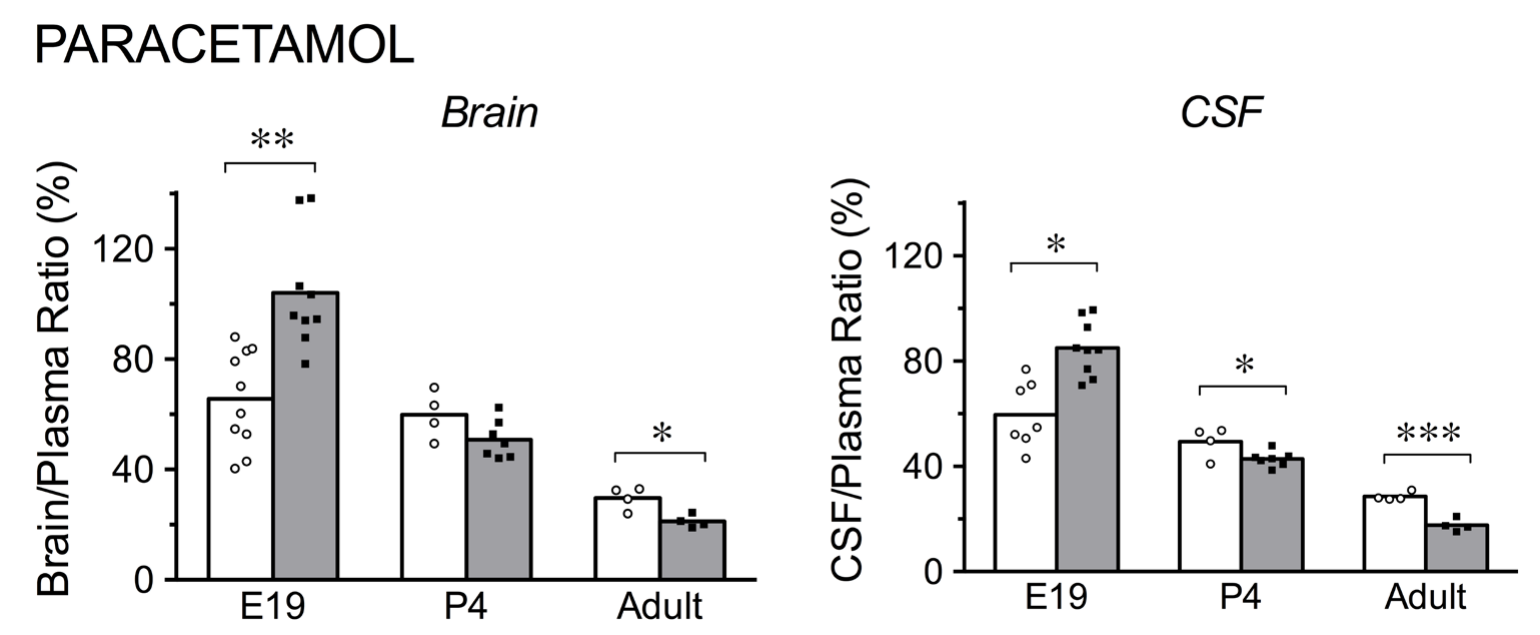
Brain/plasma and cerebrospinal fluid (CSF)/plasma concentration ratios for [ ^3^H]-paracetamol, in acute (white bars) and chronic (grey bars) experiments. Bars are group means with individual data points shown, * p<0.05, ** p<0.01, *** P<0.001. At E19, paracetamol was administered by i.p. injection to the mother. Individual fetuses were serially sampled starting at 30 minutes following maternal injection up to approximately 2.5 hours (see Figure 6 for times of sampling and maternal and fetal plasma paracetamol levels). Adult and P4 animals were injected i.p. and samples taken at 30 minutes. Note that for both acute and chronic treatment groups, the ratios were higher in brain and CSF in younger animals. At E19, following chronic doses, ratios for both brain and CSF were higher; in the adults they were lower.

### CNS drug entry

***Digoxin.*** In acute digoxin experiments ( Figure 1), the brain/plasma concentration ratio at E19 (47%) was much higher than the corresponding CSF/plasma ratio (12%). At P4, the brain/plasma (20%, p<0.05) and CSF/plasma (4%, p<0.05) ratios were both significantly lower than at E19. In the adults, the brain/plasma ratio declined further to 12% (p=0.06), while the CSF/plasma ratio remained at its already low level (4%). In chronic experiments ( Figure 1), the brain/plasma and CSF/plasma ratios were not significantly different from the acute experiments at E19 (45% and 18%, respectively) or at P4 (18% and 3%, respectively). In contrast, in adults following chronic administration, brain/plasma concentration ratio was significantly lower compared to acute experiments (5%, compared to 12%, p<0.01) and the CSF/plasma ratio also decreased significantly (3%, p<0.05).

***Cimetidine.*** In acute cimetidine experiments ( Figure 2), the brain/plasma and CSF/plasma concentration ratios were highest at E19 (56% and 54%, respectively). At P4, the brain/plasma (12%, p<0.001) and CSF/plasma (8%, p<0.001) ratios were significantly lower than at E19. In adults, the brain/plasma (13%) and CSF/plasma (12%) ratios were not statistically different from P4. There were no significant differences observed between the acute and chronic experiments for brain/plasma or CSF/plasma concentration ratios at any age.

***Paracetamol.*** In acute (single dose) experiments ( Figure 3), the brain/plasma and CSF/plasma ratios were highest at E19 (66% and 60%, respectively). At P4, ratios were lower (60% and 49%, respectively), with the CSF/plasma concentration ratio significantly different from that of E19 (p<0.05). In adults, both ratios were substantially lower than at P4 and E19, at 30% and 29%, respectively (p<0.001 for all comparisons). In chronic experiments ( Figure 3) at E19, the ratios were substantially higher than in the acute experiments for both brain (104%, p<0.01) and CSF (85%, p<0.05). At P4, ratios in chronically treated animals were lower than in acute experiments for brain (51%) and CSF (43%, p<0.05), with the CSF/plasma concentration ratio significantly different from acute data. In adults, both ratios were substantially lower following chronic treatment compared with acute experiments (brain 21%, p<0.05; CSF 18%, p<0.001).

### Gene expression of ABC efflux transporters in cerebral cortex and choroid plexus

Transcripts of all eight ABC transporters investigated were detected in brain cortex and choroid plexuses at all ages studied and in placental tissue at E19 ( Table 7). In adult brain cortex, chronic digoxin exposure resulted in a significant up-regulation of *abcb1a* (PGP) expression (1.21 fold, p<0.05). In contrast, at E19, no significant up-regulation was observed in brain cortex. Instead, chronic digoxin treatment resulted in a significant down-regulation of *abcb1b* (PGP) expression (0.63 fold, p<0.05). At P4, the response to chronic drug exposure in the brain was more variable. For all three drugs there was a down-regulation of *abcc2* (MRP2; Table 7), as well as an up-regulation of *abcg2* (BCRP) following chronic paracetamol (1.52 fold, p<0.05) and *abcc5* (MRP5) following chronic digoxin (1.15 fold, p<0.05) and chronic cimetidine (1.24 fold, p<0.01) exposure. In the choroid plexus samples at all three ages, regulation in response to chronic drug exposure appeared to be variable. Regulation was different within each age for the three drugs, as well as for each drug between ages ( Table 7). Possible correlations between ABC transporters expression and drug entry into the brain will be considered in the Discussion.

**Table 7.** Expression of ABC transporters in brain cortex, choroid plexus (lateral ventricular) and placenta (E19) in E19, P4 and adult rats, RT-qPCR. The results are fold change differences between the chronic and acute treatments. They confirm the expression of MRP1-5 ( *abcc1-5*), BCRP (abcg2) and P-glycoprotein/PGP ( *abcb1a, abcb1b*) in these rat tissues. Statistically significant differences in transcript numbers in the chronic treatment group (p<0.05) are indicated by ↑ where there was up-regulated expression of the target and by ↓ where there was down-regulated expression. * indicates a large fold change increase that did not reach statistical significance due to extremely low expression in all acute and chronically treated animals except for two animals that expressed the transporter at a high level. Note the near total absence of changes in expression in brain and choroid plexus at E19 compared to the large number of changes at P4.

	Brain Cortex								
	E19			P4			Adult		
	Paracetamol	Digoxin	Cimetidine	Paracetamol	Digoxin	Cimetidine	Paracetamol	Digoxin	Cimetidine
*abcc1*	0.95	0.99	1.00	↓ 0.73	1.06	1.11	1.00	0.89	1.20
*abcc2*	0.77	0.96	0.96	↓ 0.79	↓ 0.88	↓ 0.92	0.88	0.94	1.00
*abcc3*	0.28	0.92	0.81	0.78	1.16	1.12	0.81	0.62	1.18
*abcc4*	0.95	0.83	0.98	0.96	0.95	1.18	0.94	0.96	1.00
*abcc5*	1.49	1.13	0.97	0.92	↑ 1.15	↑ 1.24	0.91	0.90	1.11
*abcg2*	0.83	0.77	0.80	↑ 1.52	0.82	1.11	0.83	1.72	0.98
*abcb1a*	1.10	0.94	0.93	1.05	1.13	↑ 1.23	1.05	↑ 1.21	1.06
*abcb1b*	0.93	↓ 0.63	1.09	0.92	1.08	1.03	0.84	1.32	0.87
	Choroid Plexus								
	E19			P4			Adult		
	Paracetamol	Digoxin	Cimetidine	Paracetamol	Digoxin	Cimetidine	Paracetamol	Digoxin	Cimetidine
*abcc1*	1.32	1.06	1.28	↓ 0.61	1.10	0.96	0.92	0.97	1.06
*abcc2*	0.89	0.88	1.19	0.62	1.02	1.04	0.83	1.09	0.86
*abcc3*	1.28	0.91	1.46	0.66	↑ 1.17	1.05	1.14	0.85	1.12
*abcc4*	1.38	1.14	1.17	0.83	1.02	1.12	0.89	↓ 0.73	↑ 1.49
*abcc5*	1.06	1.20	1.19	0.77	↑ 1.16	0.86	0.99	0.86	1.21
*abcg2*	0.73	0.86	1.01	0.80	0.93	0.73	0.92	1.72	0.91
*abcb1a*	1.49	1.05	1.73	↓ 0.32	1.93	1.72	0.90	1.13	1.06
*abcb1b*	0.98	0.81	1.66	1.12	1.00	1.00	↑ 1.36	1.00	1.15
	Placenta								
	E19								
	Paracetamol	Digoxin	Cimetidine						
*abcc1*	↑ 1.69	↓ 0.77	↓ 0.86						
*abcc2*	0.55	0.42	*10.07						
*abcc3*	1.59	0.87	0.96						
*abcc4*	1.27	1.41	0.69						
*abcc5*	1.50	0.94	1.14						
*abcg2*	↑ 2.00	0.82	1.24						
*abcb1a*	1.52	0.78	0.86						
*abcb1b*	1.37	↓ 0.47	1.00						

### Entry of “passive” markers into the CNS

**Figure 4.**
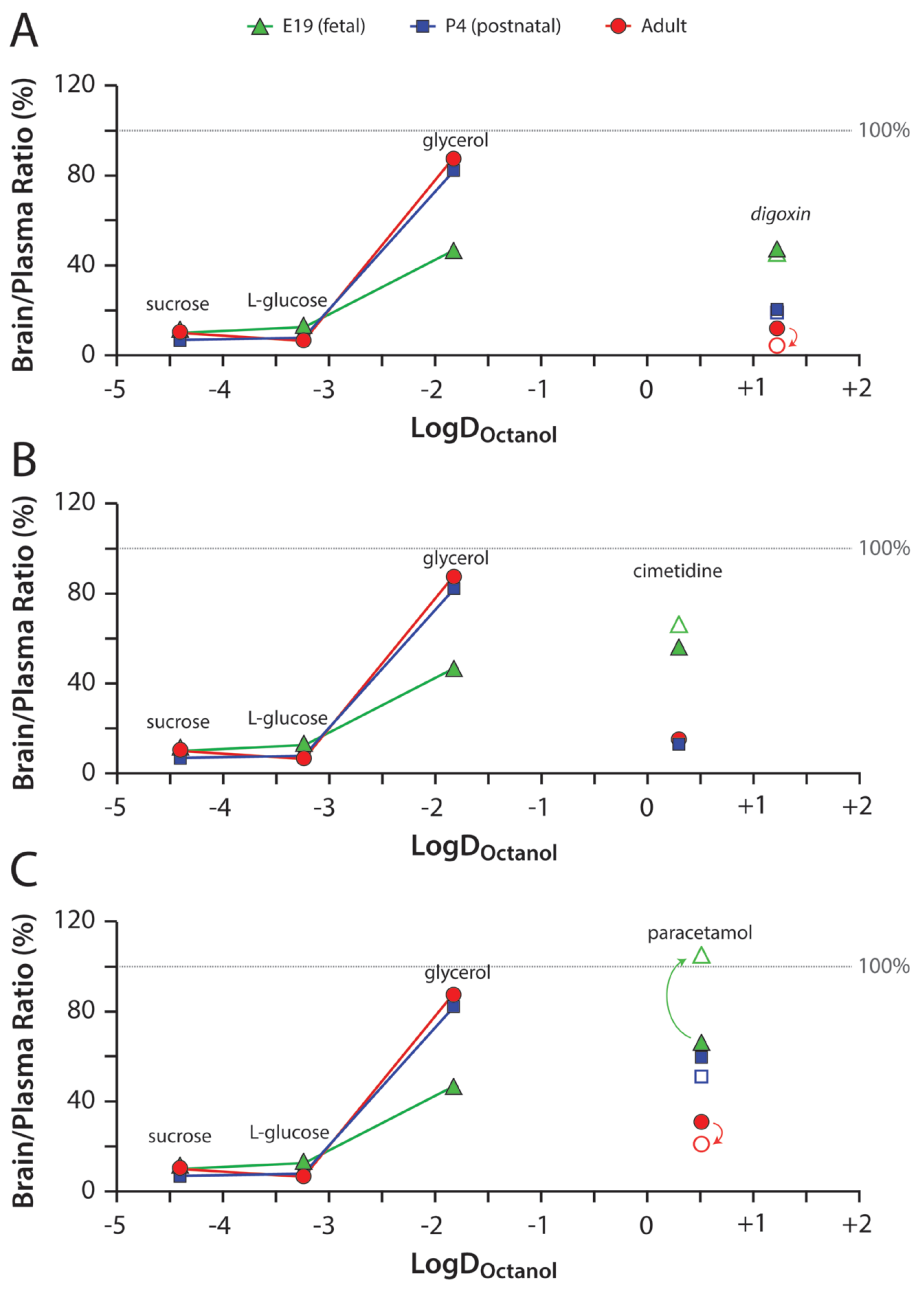
Mean brain/plasma concentration ratios for the [ ^3^H]-labelled drugs. ( **A**) digoxin, ( **B**) cimetidine and ( **C**) paracetamol in E19 fetal (green triangles), P4 postnatal (blue squares) and adult (red circles) rats plotted against their lipid solubility (LogD _Octanol_ partition coefficient) and compared with the “passive” permeability markers sucrose, L-glucose and glycerol. Filled symbols indicate acute experiments (30 minutes after IP injection) and open symbols indicate chronic experiments (after twice daily IP injections over five days). See Table 1 and Table 6 for full data and n numbers. Ratios less than 100% indicate restricted entry of the drug or marker into brain. Digoxin, despite being the most lipid soluble of the drugs was the most restricted at all ages in both the acute and chronic treatments. Cimetidine was similarly restricted at P4 and in adults, but less so at E19. Paracetamol was the least restricted of the drugs. Also note that following chronic treatment, paracetamol entry into brain decreased in adults, but was increased at E19 (direction of significant changes indicated by arrows). Chronic treatment also reduced the entry of digoxin into adult brain adults, but not at P4 and E19.

**Figure 5.**
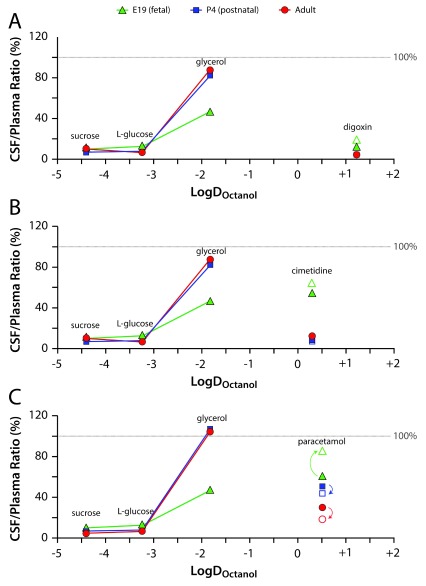
Mean CSF/plasma concentration ratios for the [ ^3^H]-labelled drugs. ( **A**) digoxin, ( **B**) cimetidine and ( **C**) paracetamol in E19 fetal (green triangles), P4 postnatal (blue squares) and adult (red circles) rats plotted against their lipid solubility (LogD _Octanol_ partition coefficient) and compared with the “passive” permeability markers sucrose, L-glucose and glycerol. Filled symbols indicate acute experiments (30 minutes after IP injection) and open symbols indicate chronic experiments (after twice daily IP injections over five days). See Table 1 and Table 6 for full data and n numbers. Ratios less than 100% indicate restricted entry of the drug or marker into CSF. Digoxin, despite being the most lipid soluble of the drugs, was the most restricted at all ages in both the acute and chronic treatments. Cimetidine was similarly restricted at P4 and in adults, but less so at E19. Paracetamol was the least restricted of the drugs. Also note that following chronic treatment, paracetamol entry into CSF decreased in adults, but was increased at E19 (direction of significant changes indicated by arrows). Chronic treatment did not significantly affect the entry of cimetidine into CSF at any of the three ages investigated.

Access of any molecule into the developing brain and CSF is determined by its physical, chemical and physiological properties and the nature of the barrier interfaces at the time (see Introduction). Therefore, a comparison was made between the drug permeability results in this study with measurements of “passive” markers of similar molecular size (L-glucose, sucrose and glycerol). These markers are thought to not bind to any influx or ABC efflux transporters and have varying lipid solubility (log D _octonol_). The brain/plasma ( Figure 4) and CSF/plasma ( Figure 5) ratios at 30 minutes after i.p. injection were determined for radiolabelled L-glucose, sucrose, and glycerol at E19, P4 and in adults. The ratios are plotted against log D _octonol_ for each marker and for each drug, with higher lipid solubility, known to have the potential to result in increased barrier permeability (see Discussion). The brain/plasma and CSF/plasma ratios of the hydrophilic markers sucrose and L-glucose were very low at all ages, whereas the ratio for the more lipophilic glycerol was about 50% at E19 (46.8% brain and CSF) and approached 100% at P4 (82.2% brain and 95% CSF) and in adults (85.5% brain and 103.9% CSF). In separate experimentation, 60-minute glycerol concentrations ratios reached approximately 80% in the brain and nearly 100% in the CSF at E19 (see Discussion), making values very similar to those of P4 and adult. The comparison of the brain/plasma ( Figure 4) and CSF/plasma ( Figure 5) ratios for the “passive” markers and the drugs at each age suggests drug exclusion, most likely by efflux transport mechanisms. By being excluded, we mean that the brain or CSF to plasma ratios are much lower than would be expected from their LogD _octonol_ position in the figure. However, this relation only applies directly to a comparison between CSF and plasma, as both compartments are water-based. The results for brain to plasma ratios can only be an indication, as both compartments are different in terms of their cellular composition; brain distribution space is a combination of intracellular and extracellular compartments.

As can be seen in the brain ( Figure 4) and in the CSF ( Figure 5), despite having the highest lipid solubility, digoxin transfer at P4 and in adults was at a level similar to the hydrophilic sucrose and L-glucose, suggesting active barrier exclusion of digoxin. If the transfer of digoxin was unobstructed, based on its logD _octonol_ value, it would be expected to reach much higher ratios at or above those for glycerol. For cimetidine, the exclusion was similar to that of digoxin at P4 and adult. In contrast, digoxin and cimetidine transfer at E19 was similar to that of glycerol. As their lipid solubility is much higher, it would be expected that, if passively transferring, their transfer ratios would be higher than glycerol; therefore, it seems likely that the degree of exclusion of digoxin and cimetidine at E19 is less than what was observed at P4 and adult. Paracetamol was generally less excluded than digoxin and cimetidine at all ages. The most striking difference was at E19 following chronic treatment, when it seems that this drug was not excluded at all (i.e. reached 100% distribution ratio between brain/CSF and plasma). In contrast to E19, the chronic treatment regime in the adult resulted in reduced entry of this drug ( Figure 4). Possible reasons for this age-related difference in paracetamol ratios are considered in the Discussion.

In summary, it is clear that the brain/plasma and CSF/plasma ratios were much higher in the E19 fetuses, both for acute and chronic experiments. The highest ratios obtained were those for paracetamol. The concentration ratios decreased substantially for all three drugs by P4, although less so for paracetamol. For this drug there was a further decrease in adult brain and CSF ratios ( Figure 1– Figure 3). This trend appeared to be specific to the lipid soluble, efflux substrate drugs and not due to general barrier permeability changes as glycerol did not follow the same trend. Entry of all three drugs into both the brain and the CSF was lower (except at E19 in chronic paracetamol experiments, see Discussion) than could be predicted from their LogD _octanol_ if their transfer was entirely unrestricted ( Figure 4 and Figure 5).

### Placental drug permeability

**Figure 6.**
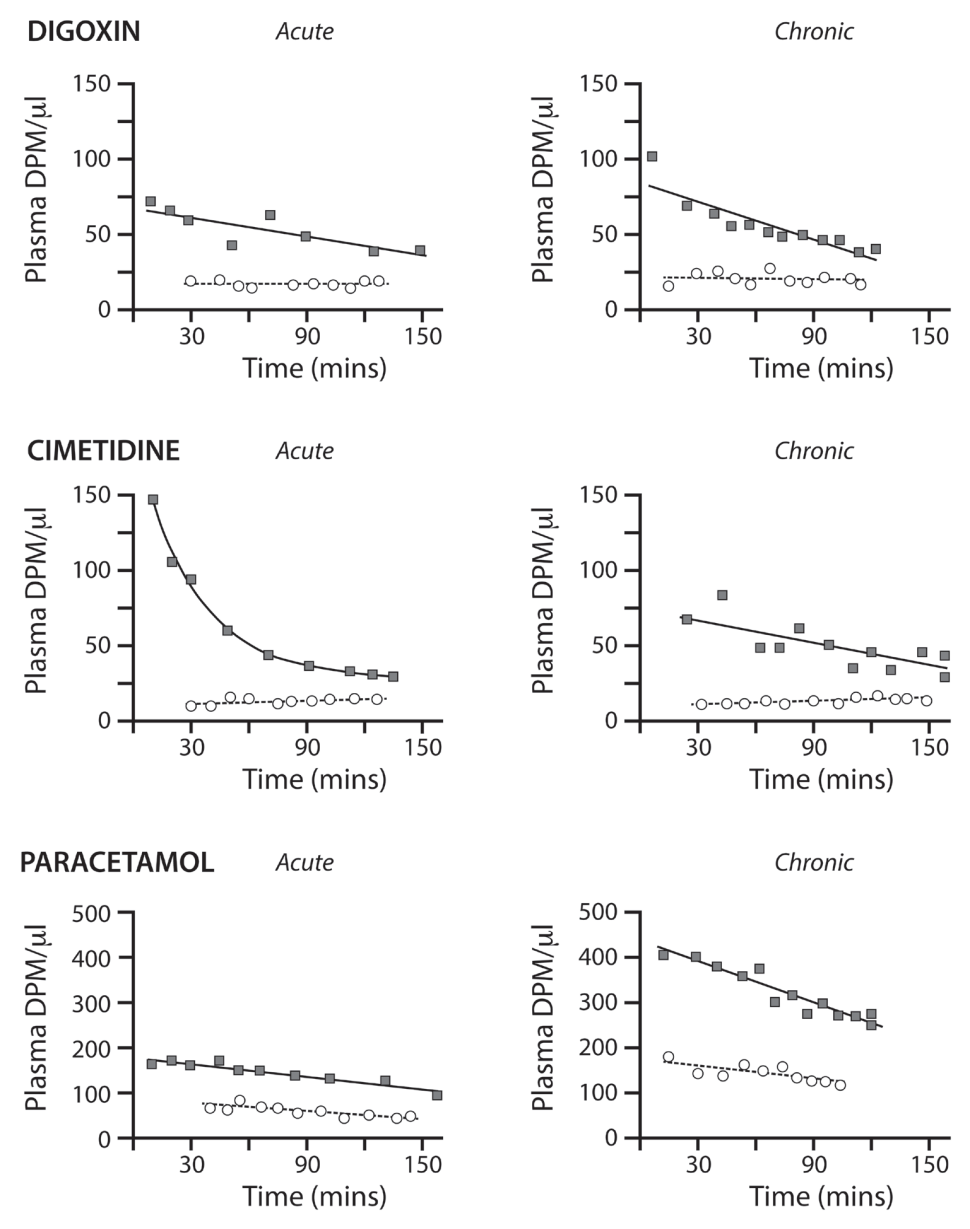
Activity levels (DPM/μl) of radiolabelled drugs (acute and chronic experiments) in E19 fetal plasma (open circles, dashed lines) and maternal plasma (filled squares, solid lines) after a single maternal i.p. injection. Note that the maternal plasma levels for both acute and chronic experiments declined progressively for all three drugs throughout the 2–2.5 hours experimental period, but the fetal plasma levels were stable for digoxin and cimetidine during the same period. The paracetamol levels in fetal plasma declined during this period. For digoxin and cimetidine, the levels of radiolabelled drugs in acute and chronic experiments were similar, but for paracetamol the levels in maternal and fetal plasma were much higher with chronic treatment. The much lower levels for each drug in fetal plasma indicates a substantial restriction of drug transfer across the placenta. Lines fitted by Least Squares Linear Regression, curve fitted by Least Squares Exponential Decay (one phase).

An estimate of the placental transfer of drug molecules at E19 was obtained by comparing the maternal and fetal plasma levels of radiolabelled drugs as illustrated in Figure 6. Maternal plasma was sampled from an arterial cannula periodically throughout the experiment (see Methods). The fetal values are from individual fetuses at the termination of their exposure to radiolabelled drug. A 2.5h post-injection cut off was established to ensure these pups were collected whilst there was good placental circulation (see Methods). As can be seen in Figure 6, the maternal plasma level of all three drugs declined throughout the experimental period but remained consistently higher than fetal plasma drug levels. Fetal plasma values followed different patterns for all three drugs. The fetal plasma paracetamol level declined with time, but at a rate that was slower compared to the maternal plasma ( Figure 6). Digoxin and cimetidine fetal plasma levels were relatively stable throughout the experiment. In order to obtain the rate of placental transfer for each drug, individual fetal plasma values were time matched to the maternal plasma samples. An average of the two nearest maternal plasma values was used where there was not a direct time match. These ratios are presented in Table 8. For all three drugs, the placenta was restricting the transfer by about 60% (paracetamol/digoxin) to 70% (cimetidine). This is indicated by the ratios of fetal to maternal plasma levels; 42% (paracetamol), 37% (digoxin) and 30% (cimetidine) ( Table 8). No significant difference was found between the transfer of the drugs across the placenta between acute and chronic administration. There were, however, some changes in placental ABC transporter expression in response to chronic treatment ( Table 7 and below).

**Table 8.** Drug and passive permeability marker transfer across the placenta at E19. Data shown are mean ± SD fetal/maternal plasma concentration ratios (%) for the radiolabelled tracers in acute (drugs and permeability markers) and chronic (drugs only) experiments. The range of the ratios obtained are shown in brackets.

	Fetal/Maternal Ratio	
Digoxin	Acute	Chronic
Mean	37.0 ± 7.6	38.6 ± 9.2
Range	(25.6 - 49.6)	(18.6 - 52.8)
n	11	12
Cimetidine	Acute	Chronic
Mean	30.3 ± 12.9	28.8 ± 10.9
Range	(10.6 - 47.7)	(14.0 - 46.9)
n	10	12
Paracetamol	Acute	Chronic
Mean	42.1 ± 6.1	43.1 ± 5.3
Range	(34.1 - 55.5)	(35.8 - 53.2)
n	11	9
L-Glucose	Acute	
Mean	16.7 ± 6.5	
Range	(8.3 - 27.7)	
n	12	
Glycerol	Acute	
Mean	106.5 ± 20.7	
Range	(68.1 - 134.7)	
n	10	

### Gene expression of ABC efflux transporters in the placenta

Despite no changes being observed in transfer of paracetamol, digoxin or cimetidine across the placenta following chronic treatment, some differences were observed in ABC-transporter expression ( Table 7). In placentas from the paracetamol treated dams, *abcc1* (MRP1) and *abcg2* (BCRP) were up-regulated (1.69 fold, p<0.05 and 2.0 fold, p<0.01 respectively). In contrast, *abcc1* (MRP1) was down-regulated following chronic treatment with digoxin or cimetidine (0.77 fold, p<0.05 and 0.87 fold, p<0.05 respectively) and *abcb1b* (PGP) was down-regulated only following digoxin treatment (0.47 fold, p<0.05).

### Transfer of “passive” markers across the placenta

**Figure 7.**
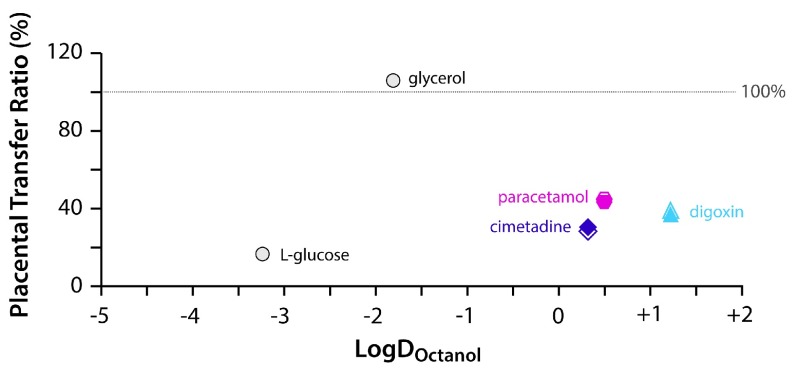
Placental transfer at E19 (fetal/maternal plasma concentration ratios) of compounds compared to their lipid solubility (LogD _Octanol_ coefficient). [ ^3^H]-labelled “passive” permeability markers L-glucose and glycerol (both open circles) are shown; along with [ ^3^H]-labelled drugs digoxin (blue triangles), cimetidine (purple diamond) and paracetamol (pink hexagon). Filled symbols indicate acute experiments and open symbols indicate chronic experiments. Values are means, see Table 8 for full data and Table 1 and Table 2 for n numbers. Note that all three drugs showed a degree of restriction (values below the trend of passive permeability) provided by the placental barrier. There was no difference between acute and chronic treatment groups for any of the drugs tested.

In the E19 animals, passive markers (L-glucose and glycerol) indicated the extent to which placenta was able to restrict passive entry of small water-soluble markers from maternal blood and into the fetal circulation ( Figure 7) during the experiments conducted. The levels of the hydrophilic marker L-glucose in the fetal plasma was low compared to the maternal plasma (16.7% ± 6.5%) during the 30 minute experimental period, whereas the more lipophilic glycerol reached 100% of the maternal plasma, indicating unrestricted transfer (Table 9).

In Figure 7, for comparative purposes, the values for the placental transfer of the three drugs are also included. Because of their high lipophilicity, it could be predicted that if they were not being actively excluded, their maternal to fetal transfer ratios would have also been close to 100%. As illustrated in Figure 7, paracetamol, digoxin and cimetidine ratios were all much lower, indicating that the placenta was able to partly impede their passage, both in acute and chronic treatment groups. Potential effects of placental exclusion on the levels of drugs reaching the fetal brains from the maternal circulation are considered in the Discussion.

## Discussion

In this study we aimed to determine the level of entry into the brain and CSF of three drugs at different ages. We also assessed the age-dependent functional capacity of brain barriers to prevent or limit the entry of potentially harmful drugs and how this may alter with a longer treatment regime. There is only limited information available about the expression and cellular distribution of key barrier defences such as ABC transporters in the developing brain, either in the human embryo/fetus ( Møllgård *et al*., 2017) or in developing rodents ( Ek *et al*., 2010). Whether these transporters are functionally active early in development or how chronic drug exposure might affect their functionality has not yet been investigated. In this paper, drug entry has been studied at fetal day 19 (E19), postnatal day four (P4) and in adult rats using clinically relevant doses of paracetamol, cimetidine and digoxin, either as a single injection or as multiple administration over five days. Well-established methods were employed to measure radiolabelled drugs/markers in the brain, blood and CSF of fetal and neonatal animals ( Dziegielewska *et al*., 1979; Dziegielewska *et al*., 1982; Habgood *et al*., 1993; Johansson *et al*., 2006; Stolp *et al*., 2005). This has been combined with RT-qPCR to determine any changes in gene expression level of the ABC transporters that have been shown to be functionally important in the adult brain (Roberts *et al*., 2008; Saidijam *et al*., 2018) in response to chronic drug treatment.

The rat has been chosen because there is considerable knowledge about brain development in this species compared with humans ( Clancy *et al*., 2001). In particular, the stage of brain development of the cerebral cortex (the main region studied) in early postnatal rats is similar to that of human fetuses at 22–24-week gestation ( Clancy *et al*., 2001). This is important because just past the mid-gestation is the earliest stage of viability for pre-term birth ( Fischer *et al*., 2009; Stoll *et al*., 2010), making the rat model noteworthy for investigating a time where the developing brain may be particularly vulnerable because of the loss of placental protection.

Permeability of the drugs into the brain and CSF was compared to that of similarly small-sized molecules, but with different diffusion coefficients and that are not actively transported, i.e. they enter the brain and CSF by “passive” diffusion only. This provides a comparison that is useful in evaluating the level of restriction of the drug’s penetration at different ages (the functionality of the relevant ABC transporter). As indicated above, the level of drug or marker attained in the brain in these experiments is a measure of “apparent permeability” rather than absolute permeability because of the influence of the turnover of CSF, which is much less in the developing brain (Saunders *et al*., 1992). Comparison of drug entry and passive marker entry allows interpretation to take account of possible effects of CSF turnover.

### Acute and chronic drug transfer to the developing brain and CSF

There are very few published studies of drug permeability of the developing brain when they were administered to pregnant rats or mice. Of these, fluoxetine and venlafaxine administered via drinking water between E0 and E10 could not be detected in the brains of the embryos ( Kaushik *et al*., 2016). All of the other drugs (digoxin, saquinavir, paclitaxel, cimetidine, apipaxaban, mitoxaurone, talinolol, carbamazepine, genisten, genisten, daidzein and coumestrol), which were administered i.v. or orally between E15 and E21, were detected in the fetal brain ( Cygalova *et al*., 2008; Enokizono *et al*., 2007; Kaushik *et al*., 2016; Petropolous *et al*., 2010; Petropolous *et al*., 2011; Saljé *et al*., 2012; Smit *et al*., 1999). However, neither fetal blood nor CSF were sampled and the brain level of the drug (usually radiolabelled) was related to maternal blood or to other fetal tissues. This makes it difficult to assess the contribution of the different barrier interfaces (placenta, blood-brain and blood-CSF barriers) that may be contributing to limiting entry of the drugs into the developing brain. The present study provides information on this, as both the maternal and fetal blood and CSF were sampled in addition to the brain tissue.

There are also very few investigations into the regulatory capacity of the developing brain in response to chronic drug exposure. Previous studies have shown that, following chronic drug challenge, blood-brain interfaces can increase their levels of protection (ABC transporters), which results in greater efflux capacity of compounds ( Cui *et al*., 2009; Hoque *et al*., 2015). Our recent study suggested that adult animals may have a greater capacity to upregulate ABC transporters at blood-brain interfaces following chronic xenobiotic exposure than early in development ( Koehn *et al*., 2019).

Digoxin has generally been found to be a substrate for PGP ( *abcb1*). Increased brain/plasma ratios for digoxin have been reported in wild type mice when co-administered with a PGP inhibitor and also when administered to *mdr1a/1b* (PGP) knockout mice, indicating that this transporter is of particular importance in limiting entry of digoxin into the brain ( Mayer *et al*., 1997). Petropoulos *et al*. (2010) suggested that levels of radiolabelled digoxin (compared to the rest of the fetus) were higher earlier in gestation when fetal levels of *abcb1a* (PGP) were lower. Smit *et al*. (1999) reported a fetal brain to maternal plasma ratio of 25% (4hrs) and 100% (24hrs) after i.v. injection of radiolabelled digoxin in pregnant mice. These would have been overestimates of the brain level, as the maternal blood level would fall progressively following injection. This type of experiment requires a dosage regime that maintains an approximately constant blood level of marker ( Dziegielewska *et al*., 1979) for a realistic estimate of the brain level to be obtained. In the present study, digoxin transferred into the adult and P4 brain (10–20%) and CSF (4%) at much lower levels compared to E19 (brain 47%, CSF 12%). The much lower entry into CSF suggests that the efflux mechanisms are more effective in the choroid plexuses than in the brain itself, even as early as E19. Following chronic treatment, transfer into the adult brain decreased (12% to 5%) in a manner that correlated with increased expression of *abcb1a* in the cerebral cortex. This result correlates with the above finding that digoxin is likely to be a PGP ( *abcb1*) substrate. As this up-regulation (and functional transfer decrease) only occurred in adults and not at P4 or E19, the results are consistent with those described by Koehn *et al*. (2019). In both studies, chronic exposure to a PGP inducer up-regulated *abcb1a* expression in the adult brain but not earlier in development, suggesting that for a range of molecular inducers, regulation of blood-brain barrier defences may be age-dependent.

Cimetidine has been typically linked to BCRP ( *abcg2*) as its efflux mechanism. Experiments in dually perfused rat placenta suggested BCRP ( *abcg2*), but not PGP ( *abcb1a/b*), involvement in cimetidine efflux ( Staud *et al*., 2006) and changes in *abcg2* (BCRP) expression in the rat brain have also been linked to differential transfer of cimetidine ( Liu *et al*., 2007). Cygalova *et al*. (2008) found that the level of radiolabelled cimetidine in the fetal brain at E18 or E21 in pregnant rats one hour after i.v. infusion was less in the older fetuses but *abcg2* (BCRP) mRNA expression was significantly less at E21 compared to E18, so presumably some other protective mechanisms may have been involved. In the present study, in acute experiments, cimetidine entry into the adult brain was similar to that of digoxin (13%) but entered the CSF to a higher ratio (12% compared to digoxin 4%). The higher degree of cimetidine transfer into the CSF compared to digoxin could be due to less ABC transporter efflux capacity at the choroid plexus or from its lower lipid solubility, allowing greater partition into the aqueous CSF fluid. There do not appear to be any cimetidine-induced changes in efflux capacity at different ages, as no differences in transfer were detected in chronically compared to acutely treated animals. Consistent with this finding are the RT-qPCR results, showing only limited changes in efflux transporter expression following chronic cimetidine treatment ( Table 7). Most changes in ABC transporter expression in response to cimetidine occurred in the brain of P4 animals. In fact, this early postnatal period of rat brain development is characterised by increased expression of some efflux transporters in response to each of the three drugs and could indicate a maturation process that is not yet fully established (see Table 7).

So far, paracetamol has not been clearly linked to any specific efflux transport mechanism, although it is known to be metabolised via glucaronidation, sulfation and (via an intermediate) glutathionation, making it likely to interact with BCRP ( *abcg2*) and the family of MRPs ( *abcc*; Mazaleuskaya *et al*., 2015). In adult rats, Courade *et al*. (2001) found brain/plasma ratios for ^3^H-paracetamol of around 40% at 45 minutes after i.v. administration in different brain regions; this is similar to our value after 30 minutes ( Figure 3). In the present study, paracetamol’s brain/plasma and CSF/plasma ratios were considerably higher than digoxin and cimetidine at E19 (66% and 60%, respectively), P4 (~60% and ~50%, respectively) and in the adult (~30% for both). This suggests that the mechanisms preventing paracetamol entry may be less effective than those targeting digoxin and cimetidine. As paracetamol has a lower lipid solubility than digoxin ( Figure 4 and Figure 5) and therefore a predicted lower barrier permeability ( Levin, 1980), this result is even more pronounced. Most interesting were the results obtained following chronic paracetamol treatment. In the adult and P4 chronically treated animals, the brain/plasma and CSF/plasma ratios decreased by approximately 10%. However, no clear regulatory mechanism could be established from RT-qPCR results, as only *abcg2* (BCRP) up-regulated at the P4 brain and *abcb1b* (PGP) up-regulated in the adult choroid plexus ( Table 7). It is therefore possible that the up-regulation occurred due to other aspects of efflux mechanisms such as the metabolising enzymes required to conjugate glucuronic acid, sulphate or glutathione groups onto paracetamol for efflux by the appropriate transporters. In fetal animals the opposite effect was observed, with an increase in both ratios to around 100%, suggesting that there was no restriction on paracetamol entry. This might indicate that brain and CSF-barrier efflux capacity was exceeded, resulting in accumulation of paracetamol in the E19 fetuses. Experiments to better understand the mechanisms regulating the entry of paracetamol into the developing brain are in progress.

### Blood-brain and blood-CSF permeability in the developing brain

The level of a drug or other marker in brain following administration depends on the duration of the experiment, its diffusion coefficient (D), its lipid solubility (Log D _octonol_), the turnover of CSF (sink effect) and the effectiveness of a specific transport mechanism if present; the latter could be inward, as in the case of amino acids and a small number of drugs, but generally outward for drugs that are substrates for ABC efflux transporters.

As has been shown in multiple studies, one major factor in the degree of the transfer is the lipid solubility of the compound. For compounds that pass barriers “passively” without active efflux, as lipid solubility increases, the transfer across the barrier interfaces should increase (Garber *et al*., 2005; Levin, 1980). The graphical data in Figure 5 and Figure 6 display each compound’s brain/plasma and CSF/plasma ratios against their lipid solubilities. For E19, P4 and adults, brain to plasma concentration ratios for passive markers (sucrose, L-glucose, glycerol) increased as lipid solubility increased.

The comparison between the brain entry of the three drugs with that of markers that do not bind to efflux transporters provides valuable insight, as most factors contributing to barrier permeability should be the same, except for active transport. The increased drug transfer earlier in development (described above) appears not to be a general property of the barrier for all molecules as glycerol did not follow this trend. This, combined with the distance of the three lipid soluble drugs below the line of passive transfer ( Figure 5 and Figure 6), indicate changes in the functional capacity of efflux transporters at the barriers over development.

### The influence of the placenta on drug transfer from maternal circulation to fetal brain

In our experiments we have been able to obtain an estimate of the placental contribution to the overall protection of the fetus from drugs administered to the pregnant mother by comparing directly the drug levels in fetal and maternal plasma. The fetal/maternal plasma ratios varied between ~30% for cimetidine treatment to ~40% for paracetamol and digoxin ( Figure 6 and Figure 7; Table 8), indicating a substantial protective barrier for these drugs provided by the placenta in late gestation in the rat. The placenta did not, however, completely prevent molecular transfer. RT-qPCR estimates of ABC transporter expression in the rat placenta at E19 confirmed the presence of *abcb1a, abcb1b, abcg2* and *abcc1-5* in varying quantities, as has been shown in several published studies ( Kalabis *et al*., 2007; Leazer *et al*., 2003; Novotna *et al*., 2004). There were only a few small changes in expression in these transporters following chronic treatment with the drugs ( Table 7). Chronic paracetamol resulted in significant up-regulation of *abcc1* (MRP1) and *abcg2* (BCRP), while digoxin and cimetidine caused a down-regulation of *abcc1*. However, these changes were not reflected in changes in placental transfer. This result may be of clinical importance as it demonstrated differences in the regulation of ABC transporters expression that are tissue specific: up-regulation with associated decreases in functional transfer into the brain, but not in the placenta.

### Comparison of maternal plasma to brain transfer in mothers and fetuses

Taking into account the level of drug exclusion provided by the placenta for all three drugs, the transfer from maternal blood to fetal brain in acute experiments was at a relatively similar concentration ratio as that from maternal blood to the maternal brain. Digoxin transfer into maternal brain was 12%, whereas the transfer from maternal blood to fetal brain was 17%. Similar results were seen for paracetamol (30% maternal brain, 28% fetal brain) and cimetidine (13% maternal brain, 17% fetal brain). In contrast to the acute experiments, different patterns of transfer were observed into the maternal and fetal brains following chronic treatment. Cimetidine, which did not up-regulate ABC transporter expression or decrease its transfer with chronic treatment, had the same ratio of transfer into both the maternal and fetal brains. Digoxin, however, decreased its entry into the maternal brain during chronic treatment, while the entry across the placenta and into the fetal brain remained the same. Paracetamol showed decreased entry into the maternal brain, while the amount entering fetal brain increased. For acute paracetamol treatment, transfer into brain (brain/maternal blood) was 30% for mother and 28% for the fetus, but after five days of twice-daily paracetamol, the transfer of a dose on the 5 ^th^ day was 21% for the mother and 45% for the fetus. Thus, in chronic treatment, paracetamol may have become less effective for the mother, but potentially more harmful for the fetus as more paracetamol reached its brain. If the same situation applies in patients, it would suggest that, where possible, duration of treatment should be as short as possible.

### Limitations of the study

This study has been restricted to two treatment conditions; namely, a single acute dose and twice daily chronic treatment over a period of five days. The latter corresponds to about one third of gestation. It is possible that with an even longer period of treatment, greater effects on ABC transporter expression correlated with greater restriction of drug entry in fetuses, neonates and adults would have been observed. We studied only one dose concentration of each drug but this was chosen to be in the clinical range. It is possible that with smaller doses, a lower rate of entry would be obtained. This could be the case particularly in our paracetamol experiments at E19; it seems likely that the greater entry of paracetamol may have been due to the capacity of the efflux transporters for this drug having been exceeded.

We have estimated drug entry using only radiolabelled drugs, rather than direct measurement of unlabelled drugs, as the amounts of fetal/postnatal fluids would have been too small for other methods such as high-performance liquid chromatography (HPLC). However, the similarity of our results in adult rats for paracetamol and those of Courade *et al*. (2001), who measured paracetamol using HPLC, suggests that the isotopically labelled drugs gave a reliable index of permeability in our experiments. There are always legitimate concerns about the extent to which results from animal studies can be extrapolated to humans. In terms of brain development, we chose P4 because of the general similarity of brain development in the rat to very preterm human infants ( Clancy *et al*., 2001). There are differences in the detail of the structure of the placentas in humans and rats, but they are both haemochorial and are much more comparable for studies such as those in this paper than, for example, the much “tighter” epitheliochorial multicotyledonary placenta of the ewe (Studert *et al*., 2011) that has been used for a lot of developmental studies.

### Conclusions and significance of the study

Our results show that at the doses used, all three drugs entered the brain at all three ages studied. The entry of all of the drugs was highest in the youngest animals (E19). This is probably a combination of the negligible turnover of CSF at this age (Saunders, 1992), allowing greater accumulation and possibly lesser activity of the relevant active efflux transporters. The entry into brain was also appreciably higher for paracetamol than the other two drugs at all three ages. The finding of entry of digoxin and cimetidine into the brain, particularly at E19, suggests that these drugs should be studied for possible long-term effects on brain development and behaviour of offspring. However, a much greater entry of paracetamol suggests that experiments to test for such effects in the offspring would be particularly appropriate, since this is the drug most commonly taken by pregnant women ( Werler *et al*., 2005). A more complex matter that requires investigation is the possibility of multiple drug administration having untoward effects due, for example, to interactions with common ABC transporter efflux mechanisms.

This study provides an experimental basis for future examination of other drugs administered in pregnancy. If the extent of drug entry into the developing brain and its regional distribution can be established, this would create a foundation for studying potential deleterious effects of drugs on brain development and possible related changes in postnatal behaviour. In addition, paracetamol and digoxin results highlight the potential differences in how adults, neonates and fetuses respond to chronic drug exposure. Adults appear more capable of responding to drug challenge by regulating ABC transporter activity, thereby allowing less drug transfer into the CSF and brain.

## Data availability

### Underlying data

Figshare: Determinants of drug entry into the developing brain: raw data files. https://doi.org/10.26188/5d3e5539dca74 ( Habgood *et al*., 2019)

This project contains the following underlying data:

- Koehn *et al*., 2019 qPCR Raw Data.xlsx
- Koehn *et al*., 2019 Sucrose Raw Data.xlsx
- Koehn *et al*., 2019 L-Glucose Raw Data.xlsx
- Koehn *et al*., 2019 Glycerol Raw Data.xlsx
- Koehn *et al*., 2019 Paracetamol Raw Data.xlsx
- Koehn *et al*., 2019 Digoxin Raw Data.xlsx
- Koehn *et al*., 2019 Cimetidine Raw Data.xlsx

Data are available under the terms of the Creative Commons Zero "No rights reserved" data waiver (CC0 1.0 Public domain dedication).

## References

[ref-1] Adedoyin A Aarons L Houston JB: Dose-dependent pharmacokinetics of cimetidine in the rat. *Xenobiotica.* 1987;17(5):595–604. 10.3109/00498258709043966 3604263

[ref-2] Australian Medicines Handbook Pty Ltd. Adelaide SA, Australia.2019 Reference Source

[ref-3] Bito LZ Bradbury MW Davson H: Factors affecting the distribution of iodide and bromide in the central nervous system. *J Physiol.* 1966;185(2):323–354. 10.1113/jphysiol.1966.sp007989 16992225 PMC1395813

[ref-4] Briggs GG Freeman RK Towers CV: Drugs in Pregnancy and Lactation: A Reference Guide to Fetal and Neonatal Risk. 11 ^th^edn. Lippincott Williams & Wilkins (LWW), Philadelphia.2017 Reference Source

[ref-5] Clancy B Darlington RB Finlay BL: Translating developmental time across mammalian species. *Neuroscience.* 2001;105(1):7–17. 10.1016/s0306-4522(01)00171-3 11483296

[ref-6] Courade JP Besse D Delchambre C: Acetaminophen distribution in the rat central nervous system. *Life Sci.* 2001;69(12):1455–1464. 10.1016/s0024-3205(01)01228-0 11531168

[ref-7] Cui YJ Cheng X Weaver YM: Tissue distribution, gender-divergent expression, ontogeny, and chemical induction of multidrug resistance transporter genes ( *Mdr1a*, *Mdr1b*, *Mdr2*) in mice. *Drug Metab Dispos.* 2009;37(1):203–210. 10.1124/dmd.108.023721 18854377 PMC2683659

[ref-8] Cygalova L Ceckova M Pavek P: Role of breast cancer resistance protein (Bcrp/Abcg2) in fetal protection during gestation in rat. *Toxicol Lett.* 2008;178(3):176–180. 10.1016/j.toxlet.2008.03.007 18450391

[ref-9] Davson H: Physiology of the Cerebrospinal Fluid. Churchill Livingston, Edinburgh.1967 Reference Source

[ref-10] Davson H Segal MB: Physiology of the CSF and blood-brain barriers. CRC press, Boca Raton.1996 Reference Source

[ref-13] Dziegielewska KM Evans CA Lai PC: Proteins in cerebrospinal fluid and plasma of fetal rats during development. *Dev Biol.* 1981;83(1):193–200. 10.1016/s0012-1606(81)80024-3 6165637

[ref-12] Dziegielewska KM Evans CA Malinowska DH: Studies of the development of brain barrier systems to lipid insoluble molecules in fetal sheep. *J Physiol.* 1979;292:207–231. 10.1113/jphysiol.1979.sp012847 490348 PMC1280854

[ref-11] Dziegielewska KM Saunders NR: Transferrin in fetal sheep cerebrospinal fluid and plasma during gestation. *Comp Biochem Physiol A Comp Physiol.* 1982;73(2):327–329. 10.1016/0300-9629(82)90079-2 6128119

[ref-14] Ek CJ Wong A Liddelow SA: Efflux mechanisms at the developing brain barriers: ABC-transporters in the fetal and postnatal rat. *Toxicol Lett.* 2010;197(1):51–59. 10.1016/j.toxlet.2010.04.025 20466047

[ref-15] Enokizono J Kusuhara H Sugiyama Y: Effect of breast cancer resistance protein (Bcrp/ *Abcg2*) on the disposition of phytoestrogens. *Mol Pharmacol.* 2007;72(4):967–975. 10.1124/mol.107.034751 17644650

[ref-16] Fischer N Steurer MA Adams M: Survival rates of extremely preterm infants (gestational age <26 weeks) in Switzerland: impact of the Swiss guidelines for the care of infants born at the limit of viability. *Arch Dis Child Fetal Neonatal Ed.* 2009;94(6):F407–F413. 10.1136/adc.2008.154567 19357122

[ref-17] Habgood MD Knott GW Dziegielewska KM: The nature of the decrease in blood-cerebrospinal fluid barrier exchange during postnatal brain development in the rat. *J Physiol.* 1993;468:73–83. 10.1113/jphysiol.1993.sp019760 8254533 PMC1143815

[ref-18] Habgood M Koehn L Huang Y: Determinants of drug entry into the developing brain: raw data files (Version 1). University of Melbourne.2019 10.26188/5d3e5539dca74 PMC6799938 31656590

[ref-19] Harrison LI Gibaldi M: Pharmacokinetics of digoxin in the rat. *Drug Metab Dispos.* 1976;4(1):88–93. 3407

[ref-20] Hoque MT Shah A More V: *In vivo* and *ex vivo* regulation of breast cancer resistant protein (Bcrp) by peroxisome proliferator-activated receptor alpha (Pparα) at the blood-brain barrier. *J Neurochem.* 2015;135(6):1113–1122. 10.1111/jnc.13389 26465636 PMC4729455

[ref-21] Johansson PA Dziegielewska KM Ek CJ: Blood-CSF barrier function in the rat embryo. *Eur J Neurosci.* 2006;24(1):65–76. 10.1111/j.1460-9568.2006.04904.x 16800861

[ref-22] Johansson PA Dziegielewska KM Liddelow SA: The blood-CSF barrier explained: when development is not immaturity. *BioEssays.* 2008;30(3):237–248. 10.1002/bies.20718 18293362

[ref-23] Kalabis GM Petropoulos S Gibb W: Breast cancer resistance protein (Bcrp1/Abcg2) in mouse placenta and yolk sac: ontogeny and its regulation by progesterone. *Placenta.* 2007;28(10):1073–1081. 10.1016/j.placenta.2007.03.010 17524480

[ref-24] Kaushik G Huber DP Aho K: Maternal exposure to carbamazepine at environmental concentrations can cross intestinal and placental barriers. *Biochem Biophys Res Commun.* 2016;474(2):291–295. 10.1016/j.bbrc.2016.04.088 27105911 PMC4891464

[ref-25] Kodaira H Kusuhara H Fujita T: Quantitative evaluation of the impact of active efflux by p-glycoprotein and breast cancer resistance protein at the blood-brain barrier on the predictability of the unbound concentrations of drugs in the brain using cerebrospinal fluid concentration as a surrogate. *J Pharmacol Exp Ther.* 2011;339(3):935–944. 10.1124/jpet.111.180398 21934030

[ref-26] Koehn LM Dziegielewska KM Møllgård K: Developmental differences in the expression of ABC transporters at rat brain barrier interfaces following chronic exposure to diallyl sulfide. *Sci Rep.* 2019;9(1):5998. 10.1038/s41598-019-42402-8 30979952 PMC6461637

[ref-27] Leazer TM Klaassen CD: The presence of xenobiotic transporters in rat placenta. *Drug Metab Dispos.* 2003;31(2):153–167. 10.1124/dmd.31.2.153 12527696

[ref-28] Levin VA: Relationship of octanol/water partition coefficient and molecular weight to rat brain capillary permeability. *J Med Chem.* 1980;23(6):682–684. 10.1021/jm00180a022 7392035

[ref-29] Lin JH Levy G: Effect of pregnancy on the pharmacokinetics of acetaminophen in rats. *J Pharmacol Exp Ther.* 1983;225(3):653–659. 6602874

[ref-30] Liu X Cheong J Ding X: Use of cassette dosing approach to examine the effects of P-glycoprotein on the brain and cerebrospinal fluid concentrations in wild-type and P-glycoprotein knockout rats. *Drug Metab Dispos.* 2014;42(4):482–491. 10.1124/dmd.113.055590 24398459

[ref-31] Liu YC Liu HY Yang HW: Impaired expression and function of breast cancer resistance protein (Bcrp) in brain cortex of streptozocin-induced diabetic rats. *Biochem Pharmacol.* 2007;74(12):1766–72. 10.1016/j.bcp.2007.08.021 17915193

[ref-32] Lyerly AD Little OM Faden R: The second wave: Toward responsible inclusion of pregnant women in research. *Int J Fem Approaches Bioeth.* 2008;1(2):5–22. 10.3138/ijfab.1.2.5 19774226 PMC2747530

[ref-33] Manautou JE de Waart DR Kunne C: Altered disposition of acetaminophen in mice with a disruption of the *Mrp3* gene. *Hepatology.* 2005;42(5):1091–1098. 10.1002/hep.20898 16250050

[ref-34] Mandal A Agrahari V Khurana V: Transporter effects on cell permeability in drug delivery. *Expert Opin Drug Deliv.* 2017;14(3):385–401. 10.1080/17425247.2016.1214565 27449574

[ref-35] Mayer U Wagenaar E Beijnen JH: Substantial excretion of digoxin via the intestinal mucosa and prevention of long-term digoxin accumulation in the brain by the mdr 1a P-glycoprotein. *Br J Pharmacol.* 1996;119(5):1038–1044. 10.1111/j.1476-5381.1996.tb15775.x 8922756 PMC1915939

[ref-36] Mayer U Wagenaar E Dorobek B: Full blockade of intestinal P-glycoprotein and extensive inhibition of blood-brain barrier P-glycoprotein by oral treatment of mice with PSC833. *J Clin Invest.* 1997;100(10):2430–2436. 10.1172/JCI119784 9366556 PMC508442

[ref-37] Mazaleuskaya LL Sangkuhl K Thorn CF: PharmGKB summary: pathways of acetaminophen metabolism at the therapeutic versus toxic doses. *Pharmacogenet Genomics.* 2015;25(8):416–426. 10.1097/FPC.0000000000000150 26049587 PMC4498995

[ref-38] Møllgård K Dziegielewska KM Holst CB: Brain barriers and functional interfaces with sequential appearance of ABC efflux transporters during human development. *Sci Rep.* 2017;7(1):11603. 10.1038/s41598-017-11596-0 28912477 PMC5599687

[ref-39] Staud F Vackova Z Pospechova K: Expression and transport activity of breast cancer resistance protein (Bcrp/Abcg2) in dually perfused rat placenta and HRP-1 cell line. *J Pharmacol Exp Ther.* 2006;319(1):53–62. 10.1124/jpet.106.105023 16809480

[ref-40] Stoll BJ Hansen NI Bell EF: Neonatal outcomes of extremely preterm infants from the NICHD Neonatal Research Network. *Pediatrics.* 2010;126(3):443–56. 10.1542/peds.2009-2959 20732945 PMC2982806

[ref-41] Stolp HB Dziegielewska KM Ek CJ: Long-term changes in blood-brain barrier permeability and white matter following prolonged systemic inflammation in early development in the rat. *Eur J Neurosci.* 2005;22(11):2805–2816. 10.1111/j.1460-9568.2005.04483.x 16324115

[ref-42] Strazielle N Ghersi-Egea JF: Efflux transporters in blood-brain interfaces of the developing brain. *Front Neurosci.* 2015;9:21. 10.3389/fnins.2015.00021 25698917 PMC4318338

[ref-43] Studdert V Gay C Blood D: Saunders Comprehensive Veterinary Dictionary. 4 ^th^Edition, Elsevier, Amsterdam, NL.2011 Reference Source

[ref-44] Taskar KS Mariappan TT Kurawattimath V: Unmasking the Role of Uptake Transporters for Digoxin Uptake Across the Barriers of the Central Nervous System in Rat. *J Cent Nerv Syst Dis.* 2017;9:1179573517693596. 10.1177/1179573517693596 28469522 PMC5392048

[ref-45] Werler MM Mitchell AA Hernandez-Diaz S: Use of over-the-counter medications during pregnancy. *Am J Obstet Gynecol.* 2005;193(3 Pt 1):771–777. 10.1016/j.ajog.2005.02.100 16150273

[ref-46] World Health Organisation: WHO guidelines on the pharmacological treatment of persisting pain in children with medical illnesses. WHO Press, Geneva.2012. 23720867

[ref-47] Wyszynski DF Shields KE: Frequency and type of medications and vaccines used during pregnancy. *Obstet Med.* 2016;9(1):21–27. 10.1177/1753495X15604099 27512486 PMC4950433

