# Supplementary material for: Determinants of drug entry into the developing brain
Source: F1000Res. 2019 Aug 7;8:1372. [Version 1] doi: 10.12688/f1000research.20078.1 (PMC6799938; doi:10.12688/f1000research.20078.1)
Supplement: Peer review history

# Peer review: Reviewer response for version 1

**DOI:** 10.5256/f1000research.22045.r52901

**Published:** 2019-09-18

**Authors:** Richard Day

**Affiliations:** 1 Department of Clinical Pharmacology & Toxicology, St Vincent's Hospital, Sydney, Australia; 2 St Vincent's Clinical School, UNSW Medicine, Sydney, Australia

The rationale for this study is the potential for transfer of medicines into the fetal and infant brain, a subject that is little understood through lack of research. It is a relevant question as there is considerable exposure of the undeveloped brain to medicines via administration to pregnant women, breast feeding women and directly to infants. The focus is upon the ABC transporter family, efficient at protecting the adult brain from lipid soluble medicines, but about which little is known in the developing brain. This question is addressed by using labelled digoxin, cimetidine and paracetamol, substrates for 3 different ABC transporters. Transport into brain and CSF is measured using radioactively labelled drug, dosed acutely or chronically and at different stages of brain development. Expression patterns of the transporters are examined at different stages of animal development and correlations sought with the transport of drugs into brain/CSF.

 There were large contrasts between E19 animals and P4/Adults for brain/plasma and CSF/plasma ratios for all three drugs, (but perhaps less so for paracetamol, especially acute administration) with E19 having greater transport/permeability. Useful information regarding ratios of marker compounds with a range of octanol-water coefficients, drug ratios and transporter expression for acute and chronic treatment was obtained. The ‘system’ showed potential for investigation of new medicines, prioritising studies of drug action on brain development on the basis of uptake into brain, e.g. paracetamol’s greater propensity for transport into brain at E19, unravelling protective effects of transporters and adaptions to componentry of the ‘system’ with dosage, duration of therapy and drug combinations. The work is well done, clearly described, well illustrated and fairly presented.

I confirm that I have read this submission and believe that I have an appropriate level of expertise to confirm that it is of an acceptable scientific standard.

# Peer review: Reviewer response for version 1

**DOI:** 10.5256/f1000research.22045.r52902

**Published:** 2019-09-09

**Authors:** Reina Bendayan

**Affiliations:** 1 Department of Pharmaceutical Sciences, Leslie Dan Faculty of Pharmacy, University of Toronto, Toronto, ON, Canada

**Summary-Study Objectives:**

****

 This is a very interesting and unique study performed by an excellent group of researchers with high expertise in the field. Overall, the study provides novel data describing the permeability of three commonly used drugs in the clinic, digoxin, cimetidine and paracetamol, into the blood, brain and CSF compartments of Sprague Dawley rats at three developmental stages. These data are further complemented by qPCR-based RNA expression studies for relevant ABC transporters in the placenta, brain and choroid plexus. The findings highlight a link between novel age-specific differences in transporter regulation and penetration of these drugs into blood, brain and CSF compartments. The work significantly advances the knowledge in the field as there is a very important lack of information related to this topic.

**Overall Comments:**

 The supporting background information described in the introduction and discussion is a very thorough representation of existing literature, and gives context to the results. Overall, the studies are carefully planned and the methods applied are robust. Limitations of the study are thoroughly described in the discussion. This work sets the stage for further investigation into the topic, and future directions are highlighted by the authors. The work significantly advances the knowledge in the field as there is a very important lack of information related to this topic.

**Specific Comments:**

  

- Additional details on the drug dosing would be helpful especially since there is ample literature describing the use of the drugs in animal models. It is clear that the dosing was adapted from the Australian Medicines Handbook; however, the authors should clarify if allometric scaling or existing literature was used to adapt the drug doses for rodent studies. The authors should also address whether the observed drug plasma concentrations in their animal model reflect clinical relevant plasma concentrations.
- The authors should consider the extent of drug plasma protein binding when they address the various factors regulating drug permeability across the various tissue compartments; only free drug will be able to permeate across biological membranes by diffusion.
- Drug concentration in biological compartments is the result of the interplay of passive diffusion across biological membranes, influx/efflux transport, and drug metabolism. While the authors have nicely characterized the role of efflux transporters, they have not fully addressed the potential role of influx transporters in the permeability of the drugs studied. Members of the Solute Carrier (SLC) superfamily could be involved in the uptake of the drugs across the various compartments. The expression of these transporters could also be very variable at the different development stages. This could be further addressed in the discussion for completeness.
      
- Measurement of radiolabeled drug in the tissue compartments will reflect total drug/metabolite concentrations rather than parent drug; hence drug metabolites may present a confounding factor in the interpretation of the data.  This should be addressed by the authors.
- In the discussion section, p17 second paragraph, please correct the spelling of the terms “gluc uronidation” “glutathionylation”.

I confirm that I have read this submission and believe that I have an appropriate level of expertise to confirm that it is of an acceptable scientific standard.

# Peer review: Reviewer response for version 1

**DOI:** 10.5256/f1000research.22045.r52190

**Published:** 2019-08-12

**Authors:** Alan Leviton

**Affiliations:** 1 Department of Neurology, Boston Children’s Hospital, Harvard Medical School, Boston, MA, USA

As I scanned this manuscript (ms), I thought the introduction rather long. On reading it, however, I was impressed how well it laid out the field succinctly, identified some of what was missing, how this report would eliminate one deficiency, and then what was found. Well done, indeed.

 The design is more than appropriate with the technically-sound assessment of multiple drugs and multiple passive permeability markers

 The methods and analysis are presented in sufficient detail for others to attempt replication. I was at first frustrated that I could not find the number of animals identified in the text. Then I found Tables 1 and 2, which, on consideration, seemed eminently reasonable given the numbers that were appropriately listed for each age group for each assessment. The authors used Tables 3 and 4 to identify materials used. Yes, the authors provided all the information needed.

 The 4 data tables (#s 5, 6, 7, and 8) and the 7 figures present all the data needed very well.

 The authors clearly document changes to support their conclusion that the functional capacities of efflux transporters at the barriers are developmentally regulated.

 I did NOT think that any revisions are called for.

I confirm that I have read this submission and believe that I have an appropriate level of expertise to confirm that it is of an acceptable scientific standard.
